# Syndromic Surveillance Tracks COVID-19 Cases in University and County Settings: Retrospective Observational Study

**DOI:** 10.2196/54551

**Published:** 2024-06-27

**Authors:** Lily Minh Wass, Derek O'Keeffe Hoare, Georgia Elena Smits, Marwan Osman, Ning Zhang, William Klepack, Lara Parrilla, Jefferson M Busche, Marin E Clarkberg, Sumanta Basu, Casey L Cazer

**Affiliations:** 1 Department of Public and Ecosystem Health, College of Veterinary Medicine, Cornell University, Ithaca, NY, United States; 2 Department of Statistics and Data Science, Cornell University, Ithaca, NY, United States; 3 Department of Neurosurgery, Yale University School of Medicine, , New Haven, Connecticut, USA; 4 GSK, Shanghai, China; 5 Tompkins County Health Department, Ithaca, NY, United States; 6 Institutional Research and Planning, Cornell University, Ithaca, NY, United States; 7 Department of Clinical Sciences, College of Veterinary Medicine, Cornell University, Ithaca, NY, United States

**Keywords:** COVID-19, epidemiology, epidemiological, SARS-CoV-2, syndromic surveillance, surveillance system, syndromic, surveillance, coronavirus, pandemic, epidemic, respiratory, infectious, predict, predictive, prediction, predictions

## Abstract

**Background:**

Syndromic surveillance represents a potentially inexpensive supplement to test-based COVID-19 surveillance. By strengthening surveillance of COVID-19–like illness (CLI), targeted and rapid interventions can be facilitated that prevent COVID-19 outbreaks without primary reliance on testing.

**Objective:**

This study aims to assess the temporal relationship between confirmed SARS-CoV-2 infections and self-reported and health care provider–reported CLI in university and county settings, respectively.

**Methods:**

We collected aggregated COVID-19 testing and symptom reporting surveillance data from Cornell University (2020‐2021) and Tompkins County Health Department (2020‐2022). We used negative binomial and linear regression models to correlate confirmed COVID-19 case counts and positive test rates with CLI rate time series, lagged COVID-19 cases or rates, and day of the week as independent variables. Optimal lag periods were identified using Granger causality and likelihood ratio tests.

**Results:**

In modeling undergraduate student cases, the CLI rate (*P*=.003) and rate of exposure to CLI (*P*<.001) were significantly correlated with the COVID-19 test positivity rate with no lag in the linear models. At the county level, the health care provider–reported CLI rate was significantly correlated with SARS-CoV-2 test positivity with a 3-day lag in both the linear (*P*<.001) and negative binomial model (*P*=.005).

**Conclusions:**

The real-time correlation between syndromic surveillance and COVID-19 cases on a university campus suggests symptom reporting is a viable alternative or supplement to COVID-19 surveillance testing. At the county level, syndromic surveillance is also a leading indicator of COVID-19 cases, enabling quick action to reduce transmission. Further research should investigate COVID-19 risk using syndromic surveillance in other settings, such as low-resource settings like low- and middle-income countries.

## Introduction

SARS-CoV-2 continues to be one of the most significant causes of morbidity and mortality, with over 697,000,000 total COVID-19 cases and 6,900,000 deaths recorded worldwide as of November 1, 2023 [1]. Although rates of COVID-19 infection, hospitalizations, and mortality have significantly declined due to global vaccination efforts [2], the emergence of the highly mutated Omicron variant and following subvariants continue to fuel spikes in COVID-19 cases [3].

National public health programs have primarily relied on diagnostic testing to gauge COVID-19 case trends. While useful for tracking the incidence of COVID-19 cases, large-scale polymerase chain reaction–based surveillance testing programs have high supply and labor costs. Coupled with the shift to at-home testing—a practice that mainly grew in the first Omicron wave beginning in December 2021—which is largely unreported [4], this has led to a substantial decline in reported daily tests in the United States. Shifting the responsibility of case reporting from health care facilities to patients has led to the underestimation of true COVID-19 trends and has challenged public health measures such as isolation and quarantine of individuals who are infected, as well as the measurement of negative effects or financial costs of COVID-19. Furthermore, May 11, 2023, marked the expiration of the US federal COVID-19 Public Health Emergency declaration, along with authorizations to collect certain public health data [5]. This triggered a pivot from COVID-19 case-based surveillance to COVID-19–associated hospital admissions as the leading indicator of COVID-19 trends, with many national COVID-19–reporting platforms like the Centers for Disease Control and Prevention (CDC) removing case-based metrics from their websites. Looking at hospitalization data as an indication of the current state of COVID-19, over 16,000 hospitalizations were reported in the week of October 15, 2023 [6].

As endemic COVID-19 seems to be the likely future of the pandemic [7], sustainable disease monitoring systems are warranted. Syndromic surveillance, or the detection and recording of symptoms before a diagnosis is confirmed, could serve as a less resource-intensive method to monitor trends in COVID-19–like illness (CLI) for public health departments and health care facilities. The National Syndromic Surveillance Project of the CDC defines CLI as “fever and cough or shortness of breath or difficulty breathing with or without the presence of a coronavirus diagnosis code. Visits meeting the CLI definition that also have mention of flu or influenza are excluded” [8].

Syndromic surveillance has already been used in a few ways throughout the pandemic. The National Syndromic Surveillance Project pivoted to reporting on CLI using local, academic, and private partnerships [9], while county health departments like Seattle and King County of Washington State have reported CLI trends from hospitalizations and emergency department (ED) visits [10]. COVID Control, a Johns Hopkins University study, piloted a symptom reporting app in 1019 counties and identified loss of taste or smell to be a leading predictor of SARS-CoV-2 rates, appearing 5 days before confirmatory diagnostic tests [11]. Limitations of these prior syndromic surveillance studies include reliance on ED data [10], which may exclude patients with COVID-19 who are asymptomatic or have mild symptoms. Although COVID-19 hospitalizations are ongoing, they have significantly declined since the onset of the pandemic and subsequent Omicron spikes, and are largely composed of populations older than 70 years [6], making surveillance that relies exclusively on ED admissions and hospitalizations biased. Additionally, the COVID Control study selected participants experiencing CLI, which does not indicate how rates of symptoms relative to the total community or population track true SARS-CoV-2 positivity rates [11]. To date, there is still limited information on how CLI surveillance can be used to accurately monitor community transmission, including asymptomatic and mild infections.

Cornell University and Tompkins County Health Department (TCHD) each developed a CLI surveillance system and a robust tracking system for confirmed COVID-19 cases. In the university setting, surveillance testing and daily symptom reporting were enforced among all individuals on campus, resulting in a daily record of testing, symptom reporting, and exposure data. In contrast, the county recorded CLI data from patients who voluntarily sought out practitioner-based care, and they had a robust free COVID-19 testing program. In this paper, we assess whether levels of reported COVID-19 symptoms are correlated with test positivity levels during the Delta and B.1 variant predominant period in a county and university setting. We identify the optimal temporal lag between changes in symptom reporting and increases in COVID-19 cases, and which symptom or health screening questions had the strongest association with test positivity rates. We provide recommendations that can be used by institutions of higher education and local county health departments to improve SARS-CoV-2 surveillance in the endemic phase of the pandemic.

## Methods

### Study Design and Population

During the 2020‐2021 academic year, Cornell University students (n=12,988 undergraduates, n=6549 graduate and professional students), faculty (n=1332), and staff (n=6739) participated in a robust COVID-19 testing program and daily syndromic surveillance, called the Daily Check. Undergraduate and professional students were tested twice a week, and graduate students, faculty, and staff were tested weekly for SARS-CoV-2 via anterior nares swab and polymerase chain reaction [12], as described elsewhere [13,14]. Supplemental SARS-CoV-2 testing occurred at the request of individuals and when increased case counts were noted in specific subpopulations. For the Daily Check, all on-campus students were asked to report daily whether they had COVID-19–like symptoms or had potential exposures to a confirmed SARS-CoV-2 case or a person with CLI. Each day, students who answered “yes” to any of these questions were labeled with a “red” status until cleared by Cornell Health with either a SARS-CoV-2 test or follow-up questions. A flowchart with the labeling logic is provided in Multimedia Appendix 1. Cornell University’s Office of Institutional Research and Planning provided aggregated data on the daily number of students, faculty, and staff who were flagged as “red,” experiencing CLI themselves, had been exposed to a confirmed COVID-19 case, had been exposed to someone experiencing CLI, and tested positive for SARS-CoV-2. The daily total number of SARS-CoV-2 tests conducted at Cornell and Daily Checks was also provided [15]. For this study, we analyzed data collected between August 17, 2020 (the first day of data collection for both the syndromic surveillance and testing programs) and February 3, 2021 (the last date before questions on the Daily Check survey changed substantially) from undergraduate students. Days with fewer than 100 SARS-CoV-2 tests conducted in the undergraduate population were excluded from the analysis.

Starting in June 2020, local health practitioners in Tompkins County were encouraged by the health department to record how many patients they saw each day and, of those patients, how many presented with CLI. TCHD defines CLI as “cough and/or shortness of breath OR at least two of the following: fever; chills; repeated shaking with chills; myalgias; headache; sore throat; new loss of sense of taste or smell.” Practices were excluded from this study if they reported fewer than 50 times between June 2020 and March 2022. A pediatric practice was excluded because the prevalence of CLI varies greatly in children relative to adults, and children also experience differing clinical presentations of COVID-19 [16,17]. Data from the remaining practices were aggregated by the date of data collection. CLI data reported on weekends were excluded because most practices were not open on the weekends. This resulted in a data set of total patient encounters, CLI encounters, and the number of practitioners who contributed to reporting each day.

Free SARS-CoV-2 testing has been available to Tompkins County residents since March 2020. A record of new positive results, cumulative total positive results, and cumulative total tests were kept between March 14, 2020, and December 20, 2022, and is publicly available on TCHD’s website [18]. As Cornell University is within Tompkins County, surveillance and diagnostic testing data from the university were included in the data set provided by Tompkins County. Starting December 1, 2020, the number of daily laboratory tests was also reported. Our analysis focused on the period from December 1, 2020 (after the number of tests was available to calculate the proportion of positive SARS-CoV-2 tests) to March 11, 2022, the last week of surveillance before the BA 2.2.12.1 variant (Omicron subvariant) exceeded more than 50% of new cases in New York State [19]. The predominant variants within our study were Delta and BA.1 [19], which minimizes possible variation due to increased transmissibility or different predominant symptoms caused by BA.2. Dates when fewer than 300 SARS-CoV-2 tests were conducted were excluded.

### Ethical Considerations

This study was determined to not meet the definition of human subject research by the Cornell University Institutional Review Board because the data were deidentified and aggregated. The original data were collected as part of public health surveillance activities by the TCHD and Cornell University, and therefore did not meet the definition of human subject research.

### Data Analysis

Within both the Cornell and TCHD data sets, the rate of SARS-CoV-2 positivity was calculated by dividing the count of new daily positive test results by the number of daily tests conducted within the undergraduate or county setting. For Cornell undergraduates, rates of CLI, exposure to CLI, and exposure to COVID-19 were calculated as the sum of each Daily Check variable over the total number of Daily Checks completed that day. The CLI rate in Tompkins County was the sum of patients experiencing CLI divided by the total number of patients seen that day. The rate of county CLI encounters on weekends was imputed as the average of Friday and Monday rates. The county CLI rate on US holidays was imputed in the same manner, by averaging rates from the two nearest dates before and after the holiday. In addition, a variable for the day of the week was encoded based on the date of data collection for both data sets.

All variables defined for the statistical analysis are detailed in Supplemental Tables 1 and 2 in Multimedia Appendix 1. For the initial descriptive analysis, the moving 7-day averages of the CLI and SARS-CoV-2 positivity rate time series were plotted. The moving average was symmetrical; the values of each variable from ±3 days and at present were summed and divided by 7.

The autocorrelation of SARS-CoV-2 positivity rates and the cross-correlation between the CLI and SARS-CoV-2 positivity time series were plotted at up to 10-day lags to identify the optimal lag period between independent variables and the outcome of SARS-CoV-2 positivity. The Granger causality test assesses whether previous values of variable *x* are useful for forecasting current values of variable *y* [20]. The grangertest function of the lmtest R package compares an autoregressive model of *y* that only uses previous values of *y* as independent variables with a model that includes previous values of *x* and *y* as independent variables [21]. We used the Granger causality test to determine if the addition of CLI rates (*x*) forecasted SARS-CoV-2 positivity rates (*y*) better than autocorrelation of *y* alone. The temporal lag of the CLI rate to be included in subsequent models was selected based on which previous days of *x* had the smallest *P* values in the Granger causality test.

Based on the findings of the autocorrelation and cross-correlation plots, two types of models were built to assess the association between CLI rates at present and from the prior 1-6 days, and the current SARS-CoV-2 positivity measure. The linear model used the SARS-CoV-2 positivity rate as the outcome. Independent variables included CLI rates at present (day 0) and from the prior 1-6 days, day of the week, and the SARS-CoV-2 positivity rate from the prior 1-6 days. In the university setting, two linear models were constructed with independent variables including either the rate of CLI or rate of exposure to CLI from up to 6 days prior. Significant variables were identified if their associated *P* value was less than .05.


$$SARS\_CoV\_2\;rate\;=\phi_{0}+\sum_{i=1}^{7}\phi_{i}CLI\;rate_{t-i+1}+\sum_{i=1}^{6}\phi_{i}SARS\_CoV\_2\;rate_{t-i}+\;weekday$$

where *i*=0, ..., *I*, and *I* is the selected maximum number of days prior.

Next, the county data was tested for overdispersion, and a negative binomial model was fitted to the data. In the negative binomial model, counts of daily SARS-CoV-2 positive tests were the outcome. CLI rates (day 0 through 6 days prior), day of the week, and counts of SARS-CoV-2 infection from the prior 1-6 days were independent variables. The CLI variable was transformed to a (0, 100) scale for the model estimates to be interpreted as percentage changes in the outcome variable of SARS-CoV-2 counts. To account for variation in the number of positive tests caused by the daily number of tests administered, an offset of the log of daily tests conducted was included in the model.


$$\log\left(SARS\_CoV\_2\;count\right)=\;\phi_{0}+\sum_{i=1}^{7}\phi_{i}log{\left(CLI\;rate\right)}_{t-i+1}\;+$$

$$\sum_{i=1}^{6}\phi_{i}\log{\left(SARS\_CoV\_2\;count\right)}_{t-i}\;+\;weekday\;+\;log\left(daily\;tests\right)$$

where *i*=0, ..., *I*, and *I* is the selected maximum number of days prior.

The significance of independent variables was assessed by comparing the *P* value associated with each input with an α of 0.05. As the negative binomial model applies a log transformation to both independent and outcome variables, the incident rate ratio estimates of the model were exponentiated to undo the log transformation.

Models within the same class were compared using the likelihood ratio test to determine whether the addition of CLI information and the day of the week yielded a significantly better fit than a model based on previous measurements of SARS-CoV-2 positivity alone.

Data were analyzed using R version 4.1.0 (R Foundation for Statistical Computing) in the RStudio environment [22].

## Results

### Analysis of CLI and Cases in University Setting

**Figure 1.**
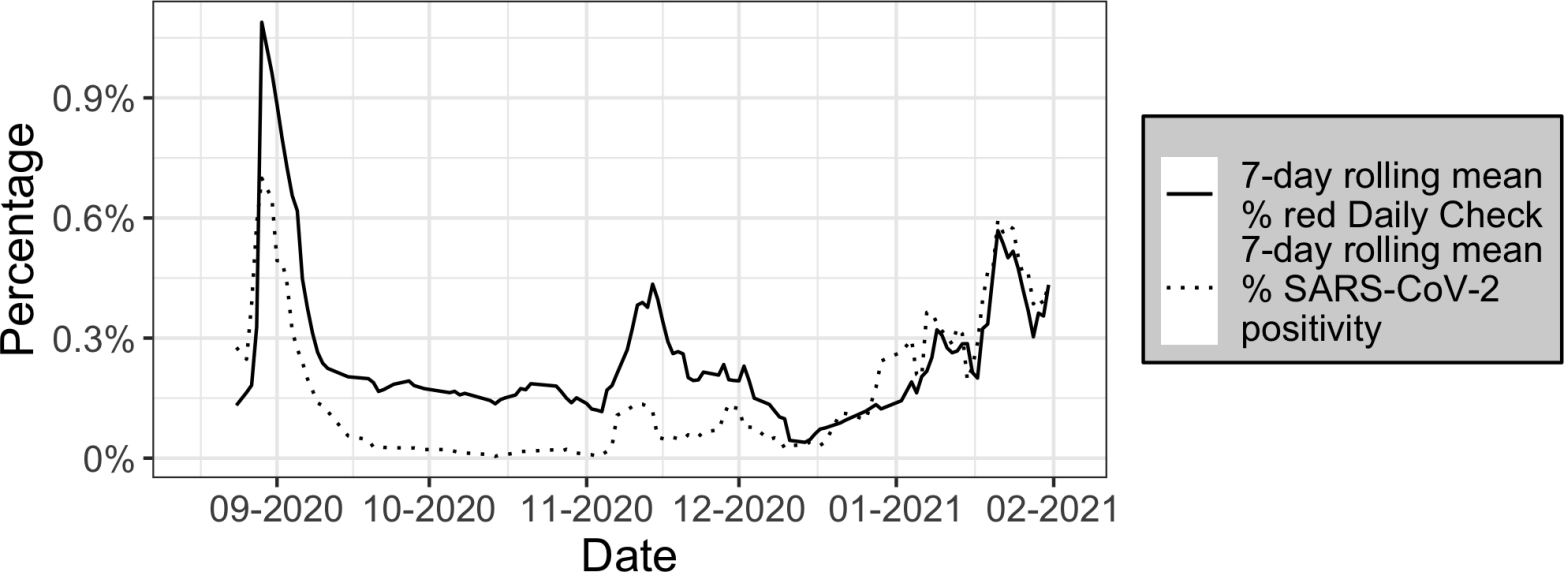
Seven-day symmetrical mean of the “red” daily check rate, shown by the solid line, and the SARS-CoV-2 test positivity rate, shown by the dotted line, from August 17, 2020, to February 3, 2021, among Cornell University undergraduate students. Date indicates day of data collection.

We collected 124 days of Daily Check and surveillance testing data between August 17, 2020, and February 3, 2021, from Cornell University. The symmetrical 7-day moving average of SARS-CoV-2 positivity rates and the rate of “red” flagged students are plotted in Figure 1.

The SARS-CoV-2 test positivity rate ranged between 0 and 0.021 with an average of 0.002. The maximum number of SARS-CoV-2 positive cases on a single day was 20. On average, 1893 tests were conducted daily. The CLI rate ranged from 0 to 0.005 with a maximum of 58 students reporting CLI in one day. The rate of exposure to CLI ranged from 0 to 0.006, with a daily maximum of 69 students reporting exposure to CLI. Finally, the rate of exposure to a confirmed COVID-19 case ranged from 0 to 0.008 with a maximum count of 87 exposures reported in one day.

The rate of SARS-CoV-2 test positivity from 1 to 4 days prior and 7 days prior was significantly autocorrelated with the SARS-CoV-2 test positivity rate at present (Supplemental Figure 1 in Multimedia Appendix 1). We observed significant cross-correlation coefficients between the present-day SARS-CoV-2 positivity rate and lagged rates of students experiencing COVID-19 symptoms, contact with someone experiencing COVID-19 symptoms, and contact with a confirmed COVID-19 case (Supplemental Figure 2 in Multimedia Appendix 1).

**Figure 2.**
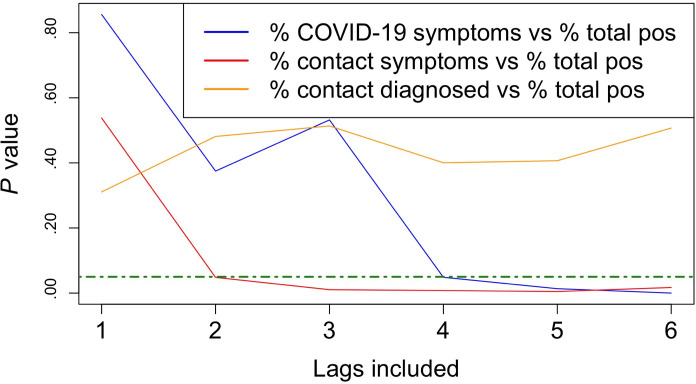
Granger causality test *P* values for models including the proportion of undergraduate students with new COVID-19 symptoms (blue), students newly in contact with people who have COVID-19 symptoms (red), and students newly in contact with a confirmed COVID-19 case (yellow) from up to 6 days prior as independent variables, with the SARS-CoV-2 positivity rate as the outcome variable, relative to the autoregressive model of the positivity rate alone. Values below the green dotted line are significant (α=.05). pos: positive test results.

The results of Granger causality test, comparing autoregression of the rate of positive tests with and without previous daily rates of CLI or exposure among undergraduates, are shown in Figure 2. The rate of COVID-19 symptoms from up to 6 days prior was found to improve forecasting of the test positivity rate relative to prior values of the positivity rate alone (*P*<.001). Similarly, including the rate of newly exposed students to CLI from up to 6 days prior significantly improved the forecasting of test positivity (*P*=.02). However, the addition of data regarding exposure to a confirmed COVID-19 case from up to 6 days prior did not significantly improve forecasting relative to an autoregression of the test positivity variable alone (*P*=.51). The results of the regressions used in the Granger test can be seen in Supplemental Tables 3-5 in Multimedia Appendix 1.

Based on the linear models, a 1 percentage point (pp) increase in the rate of undergraduates experiencing CLI was significantly associated with a 1.36 (95% CI 0.46-2.26) pp increase in the rate of SARS-CoV-2 test positivity on the same day (*P*=.003). A 1 pp increase in the rate of students reporting new contacts with people experiencing CLI was significantly associated with a 1.66 (95% CI 0.83-2.50) pp increase in the SARS-CoV-2 positivity rate on the same day as well (*P*<.001; Table 1). The full model outputs can be found in Supplemental Tables 6 and 7 in Multimedia Appendix 1.

The rate of CLI and exposure to CLI had the largest statistically significant model coefficients in their models, even compared to previous rates of SARS-CoV-2 positivity (Supplemental Tables 6 and 7 in Multimedia Appendix 1). Using the likelihood ratio test, models were found to be significantly different from the nested model that removed the time series of CLI or exposure to CLI (*P*<.001). The likelihood ratio test also showed that the addition of the day of the week did not significantly improve the rate of the CLI model (*P*=.15) but did significantly improve the exposure to the CLI model (*P*=.02) and was therefore included as an independent variable for both models.

**Table 1.** Optimal lag time between previous rates of surveillance indicators (*x*) and current SARS-CoV-2 positivity (*y*), and regression coefficient for the optimal lag. A lag time of 1 indicates COVID-19–like illness (CLI) data are taken from 1 day prior to the COVID-19 testing data, a lag of 2 is from 2 days prior, and so on.

Indicator (model type)	Optimal lag time^a^ (days)	Largest model coefficient(95% CI)	*P* value for model	*P* value for Granger causality
CLI rate among undergraduates (linear)	0	1.36 (0.46-2.26)	.003	—^b^
CLI exposure rate among undergraduates (linear)	0	1.66 (0.83-2.50)	<.001	—
CLI rate in Tompkins County (linear)	3	0.20 (0.10-0.30)	<.001	<.001
CLI rate in Tompkins County (negative binomial)	3	1.04 (1.01-1.07)	.005	<.001

a Optimal lag time is selected based on the largest model coefficient for linear models and largest incidence rate ratio for negative binomial models that is significant at α=.05.

b Granger causality assesses correlation of previous days of CLI with current measures of positivity; therefore day 0 is not included.

### Analysis of CLI and Cases in the County Setting

**Figure 3.**
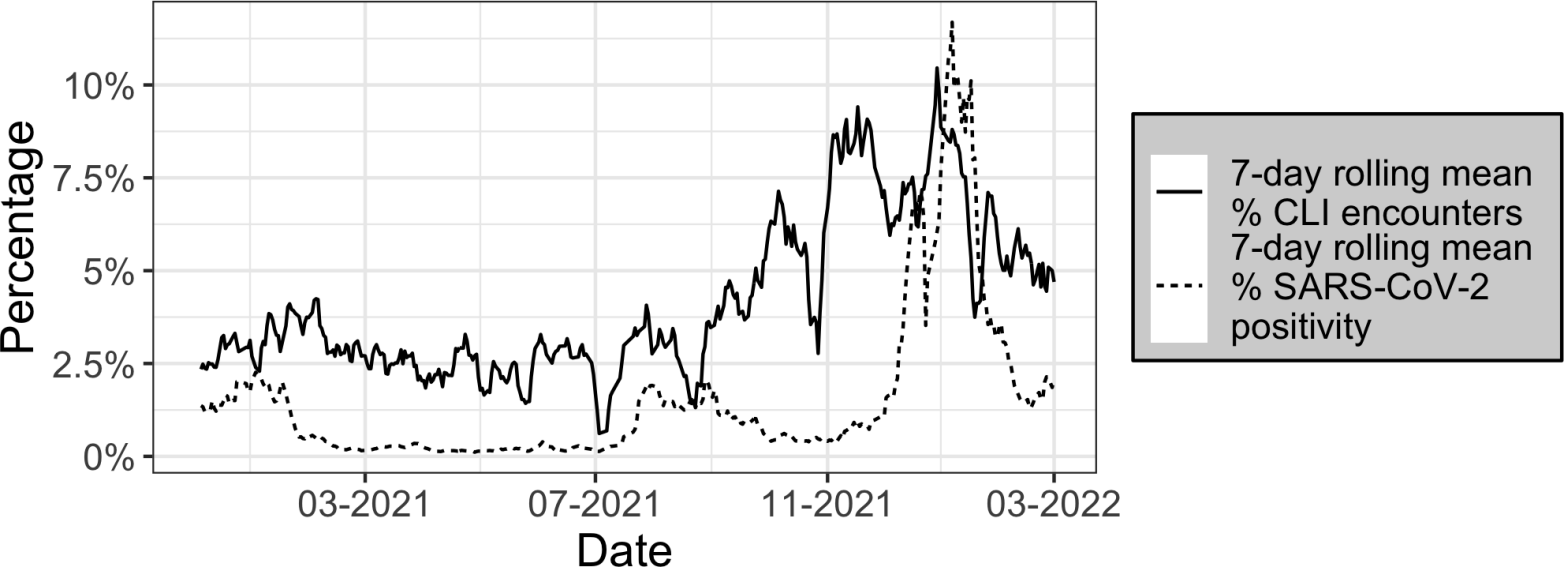
Seven-day symmetrical mean of the CLI encounter rate, shown by the solid line, and SARS-CoV-2 positivity rate, shown by the dotted line, from December 1, 2020, to March 11, 2022, in Tompkins County. CLI: COVID-19–like illness.

In the Tompkins County setting, 403 days of CLI and SARS-CoV-2 testing data between December 1, 2020, and March 11, 2022, were included after data cleaning. The 7-day moving averages of the CLI rate and SARS-CoV-2 positivity rate are plotted in Figure 3.

The SARS-CoV-2 positivity rate ranged from 0.00 to 0.64, with the maximum value observed around the peak of the first Omicron (BA.1.1) wave [23] on December 19, 2021. The average positivity rate was 0.02. The minimum daily SARS-CoV-2 positive count was 0, while the maximum was 523 cases. On average, 3235 SARS-CoV-2 tests were conducted daily. The CLI encounter (CLI encounters per total patient encounters) rate ranged from 0 to 0.71; the most patients with CLI seen on a single day was 60 with an average of 15 patients with CLI seen daily.

**Figure 4.**
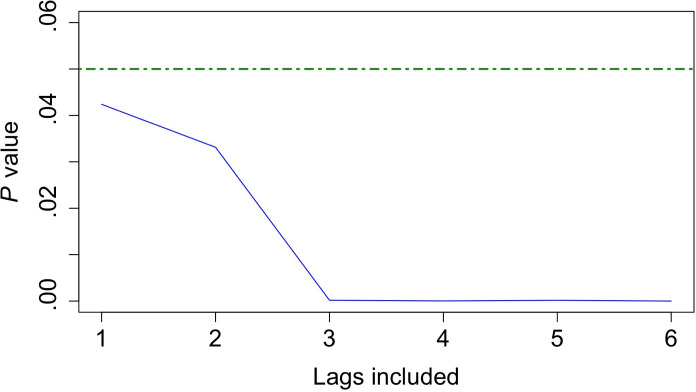
Granger causality test *P* values for a model using the COVID-19–like illness rate from 1 to 6 days prior as independent variables, with present-day SARS-CoV-2 positivity rates as the outcome variable. All values are significant at α=.05; however, *P* values decline sharply upon including lags from up to 3 or more days prior. Values below the dashed green line are significant at α=.05.

There was substantial autocorrelation of SARS-CoV-2 positivity rates; 1- through 5-day lags of the SARS-CoV-2 positivity rate had autocorrelation coefficients greater than 0.20, after which values declined (Supplemental Figure 3 in Multimedia Appendix 1). The cross-correlation plots revealed nonzero correlation coefficients between CLI rates up to 10 days prior and current rates of SARS-CoV-2 positivity (Supplemental Figure 4 in Multimedia Appendix 1). However, the highest correlation coefficients were observed in the 4-day lag (β=0.295) and 6-day lag (β=0.346) of the CLI rate. This result was confirmed by Granger causality, which revealed that including CLI rates from 3 to 6 days prior yielded the smallest *P* values (Figure 4). For CLI rates from 1 week prior (lags 1-6), minimum Granger causality *P* values were observed at a lag of 4 and 6 days (*P*<.001; Supplemental Table 8 in Multimedia Appendix 1). Based on these results, rates of CLI from 1 to 6 days prior were selected to be included in subsequent models. Previous rates of SARS-CoV-2 positivity from up to 6 days were included based on the significant autocorrelation observed (Supplemental Figure 3 in Multimedia Appendix 1). Significant autocorrelation and Granger causality of rates from 7 days prior and at present were observed, but 7-day lagged variables were not included to account for the variation that the day of the week may cause in surveillance testing and CLI reporting capabilities. Instead, the weekday variable was included to account for possible variation due to the day of the week that data was collected.

In the linear regression model (Supplemental Table 9 in Multimedia Appendix 1), a 1 pp increase in the rate of CLI 3 days prior was significantly associated with a 0.20 (95% CI 0.10-0.30) pp increase in the current SARS-CoV-2 positivity rate (*P*<.001). Similarly, a 1 pp increase in the rate of CLI 4 and 6 days prior was significantly associated with a 0.15 (95% CI 0.04-0.26) pp and 0.19 (95% CI 0.08-0.30) pp increase in current SARS-CoV-2 positivity rates (*P*=.01 and *P*<.001), respectively. The rate of CLI from up to 3 days prior had the largest regression coefficient across all linear model independent variables (Supplemental Table 8 in Multimedia Appendix 1).

In the negative binomial model (Supplemental Table 10 in Multimedia Appendix 1), a 1 pp increase in the rate of CLI encounters at a 3-day lag was significantly associated with a 4% (95% CI 1.01-1.07) increase in the number of SARS-CoV-2 positive tests at present (*P*=.005). The rate of CLI from up to 3 days prior had the highest incidence rate ratio across all numeric independent variables, including previous counts of SARS-CoV-2 positive results (Supplemental Table 10 in Multimedia Appendix 1).

Both full models were found to be significantly different from the nested model, which excluded all CLI independent variables, using the likelihood ratio test (linear model: *P*<.001; negative binomial model: *P*=.02). In addition, the likelihood ratio test also found that the inclusion of the day of the week variable significantly improves the model (linear model: *P*<.001; negative binomial model: *P*<.001). Therefore, this variable was included in the final model output (Supplemental Tables 9 and 10 in Multimedia Appendix 1).

## Discussion

### Overview

Tompkins County and the Cornell University community presented a unique opportunity to investigate the utility of CLI syndromic surveillance as an indicator of COVID-19 cases due to required testing and CLI reporting at Cornell University, in addition to widespread free testing and robust reporting of CLI by health practitioners encouraged by TCHD. We looked for congruence in rates of CLI and SARS-CoV-2 positivity to understand whether CLI trends were temporally correlated with measures of SARS-CoV-2–positive tests.

### Principal Findings

Significant autocorrelation of the SARS-CoV-2 positivity variable was observed in the university and county setting, suggesting current test positivity is influenced by levels of test positivity from earlier in the week. Cross-correlation analysis also revealed CLI to lead and lag SARS-CoV-2 rates in both settings, indicating that the two measures can offer valuable insights into each other. This reflects the expected nature of infectious disease transmission in a population without complete immunity, in which exponential growth of infections can lead to more exposures, symptoms, and positive SARS-CoV-2 tests. Given our interest in the potential use of CLI to monitor SARS-CoV-2 positivity, we demonstrate that the addition of CLI and CLI exposure data can improve efforts to assess SARS-CoV-2 positivity in low-resource settings (ie, when large-scale testing is not possible). However, the rate of exposure to confirmed COVID-19 cases was not significantly associated with the SARS-CoV-2 test positivity rate and may not be a useful measure of COVID-19 risk in universities. A possible explanation for COVID-19 exposure not being a significant variable in our analysis is that confirmed exposure to a COVID-19 case is dependent on testing capacity to deliver a quick test result. In the university’s syndromic surveillance, students could have already been flagged as “red” based on reporting exposure to CLI before a positive test result has confirmed the case. Depending on testing capacity and availability, possible exposure to COVID-19 could be a stronger variable than confirmed exposure to COVID-19 for tracking SARS-CoV-2 in real time.

In the university setting, linear models demonstrated that reporting of CLI and exposure to CLI among students track changes in the SARS-CoV-2 positivity rate in real time. Within Tompkins County, both the linear and negative binomial model indicated that the strongest variable associated with present-day SARS-CoV-2 positivity is the rate of CLI from up to 3 days prior. This discrepancy in the lag time of the CLI indicator could reflect the difference in adherence to the two programs. The university surveillance program was strictly enforced for the entire on-campus population and may have been able to identify changes in SARS-CoV-2 cases immediately. By comparison, the testing and CLI reporting of Tompkins County was voluntary. This may have caused lags between when symptoms appeared and diagnosis by a positive test result due to delays in testing based on appointment availability, patients’ work schedules, access to transportation, and other conflicting factors. Cornell University demonstrates how mandatory symptom and exposure reporting could track SARS-CoV-2 positivity in real time. However, with mandatory surveillance programs being unrealistic to implement, Tompkins County illustrates how the utilization of existing infrastructure like local practitioners is also able to effectively measure CLI, which is associated with cases. Practitioners were asked to count CLI cases each day, a process that could be applied to larger practitioner and hospital networks. We demonstrated that CLI rates from practitioner-based surveillance track SARS-CoV-2 positivity rates with a small lag. Trends such as spikes in COVID-19 identified from syndromic surveillance programs could then be validated by brief surveillance testing or distribution of at-home tests.

### Limitations

The study findings should be carefully interpreted within the context of the study population. Cornell University is located in a small county in upstate New York, with a population of 105,162 in 2021 [24]. Tompkins County is somewhat isolated geographically, so these results may not generalize to denser urban areas or more rural areas. While influenza rates substantially decreased during the 2020‐2021 flu season [25], some presentations of CLI in the study population are inevitably due to flu and other respiratory illnesses. This may explain a spike in university cases of CLI during November and December 2021 that was not accompanied by an increase in SARS-CoV-2 positivity, as flu rates did increase in New York State during this period [26]. On the other hand, given that both flu seasons included in our study period were less severe than usual, it is possible that the associations we observed may not be as strong when other common respiratory illnesses are in wider circulation. In addition, participating practitioners of our study voluntarily partook in syndromic surveillance without reimbursement. To apply this model of surveillance on a larger scale, financial incentives should be considered to ensure robust reporting—a cost that would need to be considered before implementation. Finally, as this was an observational study, we can only infer relationships and not their underlying cause.

### Significance

Our findings are contextualized by the shift away from robust SARS-CoV-2 testing and reporting in the United States. At-home tests are now commonly used and typically are not included in COVID-19 case count reporting [4]. Federal funding for SARS-CoV-2 testing is diminishing [27], and COVID-19 response teams of health departments are being scaled back. On May 11, 2023, the US federal government ended the COVID-19 Public Health Emergency declaration [5]. As a result, the CDC and other reporting platforms no longer publish metrics related to COVID-19 community transmission like case counts [5]. This significantly limits the ability of health departments to identify and mitigate increases in cases. Low-resource alternatives like syndromic surveillance could help to fill this gap in our knowledge of SARS-CoV-2 local transmission and build on the currently favored methods of hospital-based surveillance. Alternatives to resource-intensive testing are especially important for detecting outbreaks in low-resource settings where diagnostic testing infrastructure is poor [28,29]. This includes low- and middle-income countries as well as impoverished communities in the United States.

### Conclusions

Finally, we emphasize that the estimates of our regressions, both linear and negative binomial, should not necessarily be interpreted as definite predicted changes in the rate of SARS-CoV-2. Our goal was not to build a predictive model but to instead investigate associations between the outcome, current levels of SARS-CoV-2 cases and test positivity, and recent measures of CLI. We focused on the statistically significant lagged CLI rate variables that demonstrate changes in CLI rates can lead SARS-CoV-2 rates by a few days, as confirmed by both the negative binomial and linear models in the county setting. The university setting models the advantage of an enforced daily health questionnaire whose measures of CLI and exposure to CLI appear to track on-campus SARS-CoV-2 positivity in real time. The strongest takeaway from our results is that the two variables, CLI and SARS-CoV-2 positivity, are correlated, and CLI should be further explored as a low-resource way to monitor the risk of SARS-CoV-2 in the absence of robust testing. Our results support the push to integrate CLI symptom reporting into the routine services of health practitioners as a potentially easy and cost-effective approach to monitoring SARS-CoV-2 transmission. This forms a foundation for future research that should further characterize the relationship between COVID-19 symptoms and SARS-CoV-2 positivity rates.

## Supplementary material

10.2196/54551 Multimedia Appendix 1 Supplementary materials.

## Abbreviations

CDC: Centers for Disease Control and Prevention
CLI: COVID-19–like illness
ED: emergency department
pp: percentage point
TCHD: Tompkins County Health Department
