# Supplementary material for: Syndromic Surveillance Tracks COVID-19 Cases in University and County Settings: Retrospective Observational Study
Source: JMIR Public Health Surveill. 2024 Jun 27;10:e54551. doi: 10.2196/54551 (PMC11220726; doi:10.2196/54551)
Supplement: Multimedia Appendix 1 [file publichealth-v10-e54551-s001.docx]

# **Supplemental**

| **Variable** | **Definition** |
| --- | --- |
| Date | Date of data collection |
| UG_DC_Total | Daily count of undergraduates who submitted to the Daily Check |
| UG_New_Red | Daily count of undergraduates flagged as “red” by the Daily Check that day |
| UG_COVID_NewRed | Daily count of undergraduates who reported experiencing CLI |
| UG_contact_symp_NewRed | Daily count of undergraduates who reported recently coming into contact with someone experiencing CLI |
| UG_contact_diag_NewRed | Daily count of undergraduates who reported recently coming into contact with a confirmed COVID-19 case |
| total_pos | Daily count of new SARS-CoV-2 positive test results among undergraduates |
| total_pos_surv | Daily count of SARS-Cov-2 positive tests from surveillance testing |
| total_pos_diag | Daily count of SARS-Cov-2 positive tests from diagnostic testing |
| total_tests | Daily count of SARS-CoV-2 tests conducted among undergraduates |
| UG_Surv | Daily count of SARS-CoV-2 surveillance tests conducted among undergraduates |
| UG_Diag | Daily count of SARS-CoV-2 diagnostic tests conducted among undergraduates |
| pos_rate | total_pos/Total Undergrad |
| CLI_rate | UG_New_Red/UG_DC_Total |
| weekday | Day of the week |
| case_Lag1:case_Lag6 | total_pos from 1 to 6 days prior |
| pos_Lag1:pos_Lag6 | pos_rate from 1 to 6 days prior |
| CLI_Lag1:CLI_Lag6 | CLI_rate from 1 to 6 days prior |

**Supplemental Table 1**. Description of variables in the Cornell undergraduate dataset

| **Variable** | **Definition** |
| --- | --- |
| date_collect | Date of data collection |
| sum_practitioners | Daily count of practitioners who submitted to daily reporting |
| sum_CLI | Daily count number of CLI patients encountered |
| sum_patients | Daily count number of patients seen |
| daily_test_eclrs | Total number of SARS-CoV-2 tests conducted based on NYS Electronic Clinical Laboratory Reporting System |
| new_pos | Daily count of new SARS-CoV-2 positive test results |
| variant | Dominant SARS-CoV-2 variant circulating at this date |
| weekday | Day of the week |
| pos_rate | new_pos/daily_test_eclrs |
| CLI_rate | sumCLI/sumPatients |
| case_Lag1:case_Lag6 | new_pos from 1 to 6 days prior |
| pos_Lag1:pos_Lag6 | pos_rate from 1 to 6 days prior |
| CLI_Lag1:CLI_Lag6 | CLI_rate from 1 to 6 days prior |

**Supplemental Table 2.** Description of variables in Tompkins County dataset.

| **Lag (Days) of COVID-19 symptoms** | **P-value** |
| --- | --- |
| 1 | .86 |
| 2 | .38 |
| 3 | .53 |
| **4** | **.049** |
| **5** | **.014** |
| **6** | **<.001** |

**Supplemental Table 3.** Results of Granger causality test assessing previous daily rates of undergraduate COVID-19 symptoms in the forecasting of present-day SARS-CoV-2 positivity rate. Bolded lags are significant at α=.05. The null hypothesis that rates of COVID symptoms are not associated with rates of SARS-CoV-2-positivity can be rejected for lags day four through seven.

| **Lag (Days) of exposure to CLI** | **P-value** |
| --- | --- |
| 1 | .54 |
| **2** | **.048** |
| **3** | **.011** |
| **4** | **.008** |
| **5** | **.005** |
| **6** | **.02** |

**Supplemental Table 4.** Results of Granger causality test assessing previous daily rates of undergraduate exposure to contacts with CLI in the forecasting of present-day SARS-CoV-2 rates. Bolded lags are significant at α=.05. The null hypothesis that rates of exposure to contacts with COVID-19 symptoms is not associated with SARS-CoV-2 positivity rates can be rejected at lags two through seven.

| **Lag (Days) of exposure to CLI** | **P-value** |
| --- | --- |
| 1 | .31 |
| 2 | .48 |
| 3 | .51 |
| 4 | .40 |
| 5 | .41 |
| 6 | .51 |

**Supplemental Table 5.** Results of Granger causality test assessing previous daily rates of student exposure to confirmed COVID-19 cases in the forecasting of present-day SARS-CoV-2-positivity rates. No lags were found to be significantly associated with present day positivity rates at α=.05.

| **Predictor** | **Estimate** | **95% CI** | **P-value** |
| --- | --- | --- | --- |
| **pos_Lag1** | **0.29** | **(0.10, 0.48)** | **.003** |
| pos_Lag2 | -0.02 | (-0.22, 0.18) | .83 |
| pos_Lag3 | 0.13 | (-0.07, 0.33) | .20 |
| pos_Lag4 | 0.17 | (-0.03, 0.37) | .10 |
| pos_Lag5 | 0.13 | (-0.08, 0.34) | .21 |
| pos_Lag6 | 0.12 | (-0.09, 0.32) | .26 |
| **COVID_symp_Lag0** | **1.36** | **(0.46, 2.26)** | **.003** |
| COVID_symp_Lag1 | 0.37 | (-0.48, 1.23) | .39 |
| COVID_symp_Lag2 | 0.35 | (-0.46, 1.17) | .39 |
| COVID_symp_Lag3 | -0.59 | (-1.41, 0.24) | .16 |
| **COVID_symp_Lag4** | **-2.29** | **(-3.10, -1.48)** | **<.001** |
| COVID_symp_Lag5 | -0.43 | (-1.16, 0.31) | .25 |
| COVID_symp_Lag6 | 0.40 | (-0.32, 1.12) | .27 |
| Monday | 1 x 10-5 | (-2 x 10^-3^, 2 x 10^-3^) | .99 |
| Tuesday | -9 x 10-4 | (-3 x 10^-3^, 1 x 10^-3^) | .37 |
| Wednesday | -3 x 10-4 | (-2 x 10^-3^, 2 x 10^-3^) | .78 |
| Thursday | -2 x 10-3 | (-3 x 10^-3^, 9 x 10^-4^) | .26 |
| Saturday | -2 x 10-3 | (-4 x 10^-3^, 3 x 10^-4^) | .09 |
| Sunday | 8 x 10-4 | (-1 x 10^-3^, 3 x 10^-3^) | .47 |

**Supplemental Table 6.** Summary of undergraduate setting linear model using CLI rate to predict SARS-CoV-2 positivity rate. Bolded: *P*<.05. Outcome: SARS-CoV-2 test positivity rate. Predictors: lagged proportion of students with COVID-19 symptoms, lagged positive test rate.

| **Predictor** | **Estimate** | **95% CI** | **P-value** |
| --- | --- | --- | --- |
| **pos_Lag1** | **0.25** | **(0.04, 0.47)** | **.02** |
| pos_Lag2 | -0.03 | (-0.25, 0.19) | .79 |
| **pos_Lag3** | **0.29** | **(0.06, 0.52)** | **.010** |
| **pos_Lag4** | **0.23** | **(-3 x 10-3, 0.45)** | **.047** |
| pos_Lag5 | 0.07 | (-0.15, 0.29) | .53 |
| pos_Lag6 | 0.02 | (-0.20, 0.24) | .86 |
| **contact_symp_Lag0** | **1.66** | **(0.83, 2.50)** | **<.001** |
| contact_symp_Lag1 | 0.18 | (-0.71, 1.06) | .70 |
| **contact_symp_Lag2** | **-1.81** | **(-2.70, -0.93)** | **<.001** |
| contact_symp_Lag3 | -0.51 | (-1.47, 0.44) | .29 |
| contact_symp_Lag4 | -0.72 | (-1.66, 0.23) | .13 |
| contact_symp_Lag5 | 0.21 | (-0.72, 1.13) | .66 |
| contact_symp_Lag6 | 0.11 | (-0.67, 0.89) | .78 |
| Monday | 8 x 10-4 | (-1 x 10^-3^, 3 x 10^-3^) | .45 |
| Tuesday | -6 x 10-4 | (-3 x 10^-3^, 1 x 10^-3^) | .58 |
| Wednesday | -4 x 10-4 | (-3 x 10^-3^, 2 x 10^-3^) | .74 |
| **Thursday** | **-2 x 10-3** | **(-5 x 10^-3^, -3 x 10^-4^)** | **.03** |
| Saturday | -9 x 10-4 | (-3 x 10^-3^, 1 x 10^-3^) | .44 |
| Sunday | 8 x 10-4 | (-2 x 10^-3^, 3 x 10^-3^) | .51 |

**Supplemental Table 7.** Summary of undergraduate linear model using rate of exposure to CLI as predictor of SARS-CoV-2 positivity rate. Bolded: *P* <.05. Outcome: SARS-CoV-2-test positivity rate. Predictors: lagged proportion of students exposed to someone with COVID-19 symptoms, lagged positive test rate, day of week.

| **Lag (Days) of exposure to CLI** | **P-value** |
| --- | --- |
| **1** | **.04** |
| **2** | **.03** |
| **3** | **<.001** |
| **4** | **<.0001** |
| **5** | **<.001** |
| **6** | **<.0001** |

**Supplemental Table 8.** Results of Granger causality test assessing previous daily rates of countywide CLI in forecasting present day SARS-CoV-2 positivity rate. Bolded lags are significant at α=0.05. The null hypothesis that rates of COVID symptoms are not associated with rates of SARS-CoV-2-positivity can be rejected for lags day one through seven.

| **Predictor** | **Estimate** | **95% CI** | **P-value** |
| --- | --- | --- | --- |
| pos_rate_Lag1 | 0.021 | (-0.07, 0.12) | .66 |
| **pos_rate_Lag2** | **0.11** | **(0.02, 0.21)** | **.02** |
| pos_rate_Lag3 | 0.04 | (-0.06, 0.13) | .42 |
| pos_rate_Lag4 | 0.07 | (-0.03, 0.16) | .16 |
| pos_rate_Lag5 | 0.10 | (-0.01, 0.21) | .07 |
| **pos_rate_Lag6** | **0.12** | **(0.01, 0.23)** | **.03** |
| CLI_Lag0 | 0.02 | (-0.11, 0.15) | .77 |
| CLI_Lag1 | -0.12 | (-0.26, 0.02) | .08 |
| CLI_Lag2 | -0.03 | (-0.15, 0.08) | .55 |
| **CLI_Lag3** | **0.20** | **(0.10, 0.30)** | **<.001** |
| **CLI_Lag4** | **0.15** | **(0.04, 0.26)** | **.010** |
| CLI_Lag5 | 0.01 | (-0.10, 0.13) | .85 |
| **CLI_Lag6** | **0.19** | **(0.08, 0.30)** | **<.001** |
| Monday | 0.01 | (-0.01, 0.02) | .37 |
| Tuesday | 0.01 | (-0.01, 0.02) | .33 |
| Wednesday | 2 x 10-3 | (-0.01, 0.02) | .81 |
| Thursday | 2 x 10-3 | (-0.01, 0.02) | .78 |
| Saturday | 2 x 10-3 | (-0.01, 0.02) | .83 |
| **Sunday** | **0.06** | **(0.04, 0.08)** | **<.001** |

**Supplemental Table 9.** Summary of county setting linear model. Bolded: *P*<.05. Outcome: SARS-CoV-2 test positivity rate. Predictors: lagged positive test rate, lagged proportion of CLI patients, day of week.

| **Predictor** | **IRR** | **95% CI** | **P-value** |
| --- | --- | --- | --- |
| **caseLag1** | **1.00** | **(1.00, 1.01)** | **.02** |
| **caseLag2** | **1.00** | **(1.00, 1.01)** | **.010** |
| caseLag3 | 1.00 | (1.00, 1.01) | .22 |
| caseLag4 | 1.00 | (1.00, 1.00) | .84 |
| caseLag5 | 1.00 | (1.00, 1.01) | .09 |
| caseLag6 | 1.00 | (1.00, 1.00) | .24 |
| CLI_Lag0 | 1.01 | (0.98, 1.05) | .51 |
| CLI_Lag1 | 0.98 | (0.94, 1.03) | .22 |
| CLI_Lag2 | 1.01 | (0.98, 1.05) | .48 |
| **CLI_Lag3** | **1.04** | **(1.01, 1.07)** | **.005** |
| CLI_Lag4 | 1.01 | (0.98, 1.05) | .32 |
| CLI_Lag5 | 0.99 | (0.95, 1.03) | .40 |
| CLI_Lag6 | 1.01 | (0.98, 1.04) | .63 |
| **Monday** | **1.60** | **(1.07, 2.40)** | **.02** |
| **Tuesday** | **1.68** | **(1.14, 2.47)** | **.008** |
| Wednesday | 1.17 | (0.80, 1.70) | .41 |
| Thursday | 1.21 | (0.84, 1.74) | .31 |
| Saturday | 1.03 | (0.71, 1.50) | .87 |
| **Sunday** | **3.49** | **(2.22, 5.57)** | **<.001** |

**Supplemental Table 10.** Summary of county setting negative binomial model. Bolded: *P*<.05. Outcome: counts of SARS-CoV-2-positive test results; offset by total tests. Predictors: lagged positive test count, lagged proportion of CLI patients, day of week. IRRs of all predictor variables are exponentiated to undo log transformation.


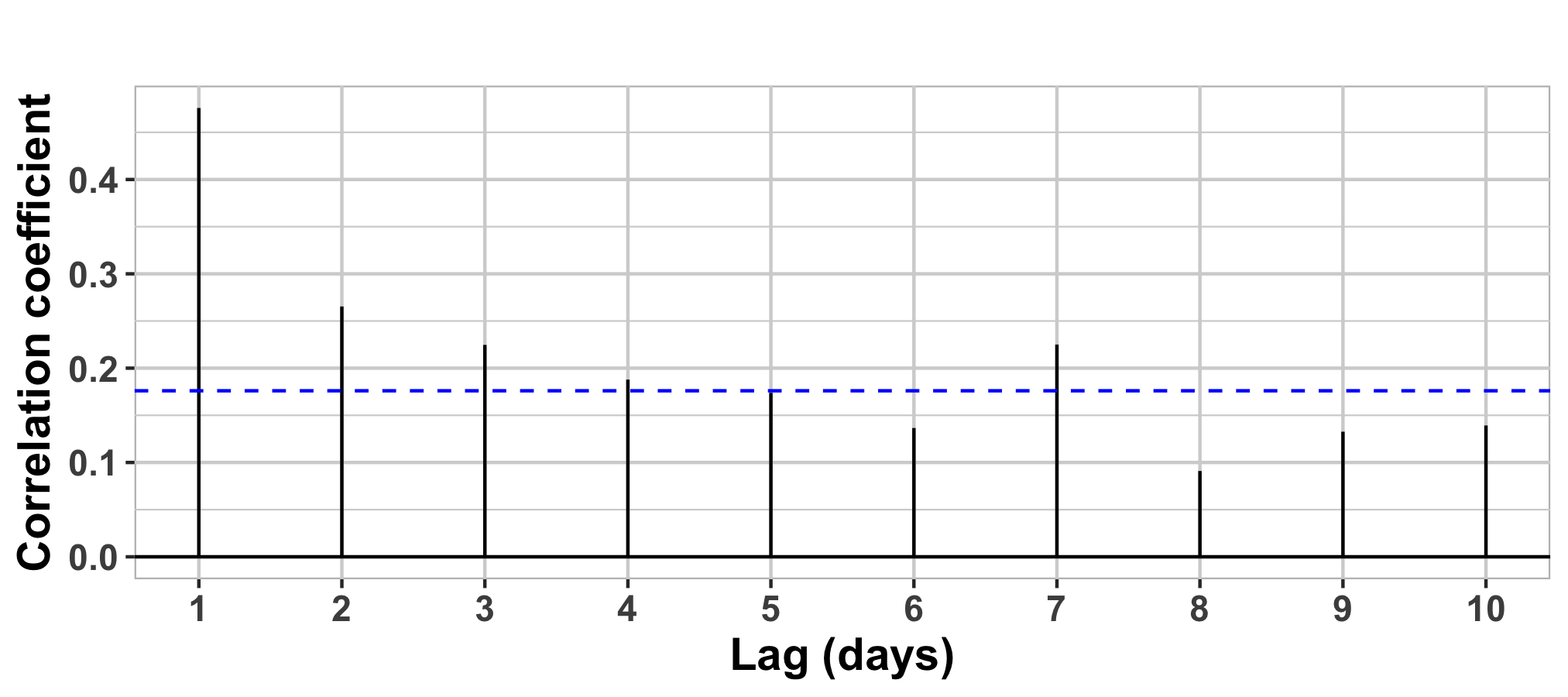


**Supplemental Figure 1.** Autocorrelation plot of correlation coefficients between SARS-CoV-2-positivity rate among Cornell undergraduates and the same variable measured at days 1-10 prior. Correlation coefficients above the dashed blue lines indicate values are significantly different from zero. Significant autocorrelation observed lags of one through four- and seven-day lags.


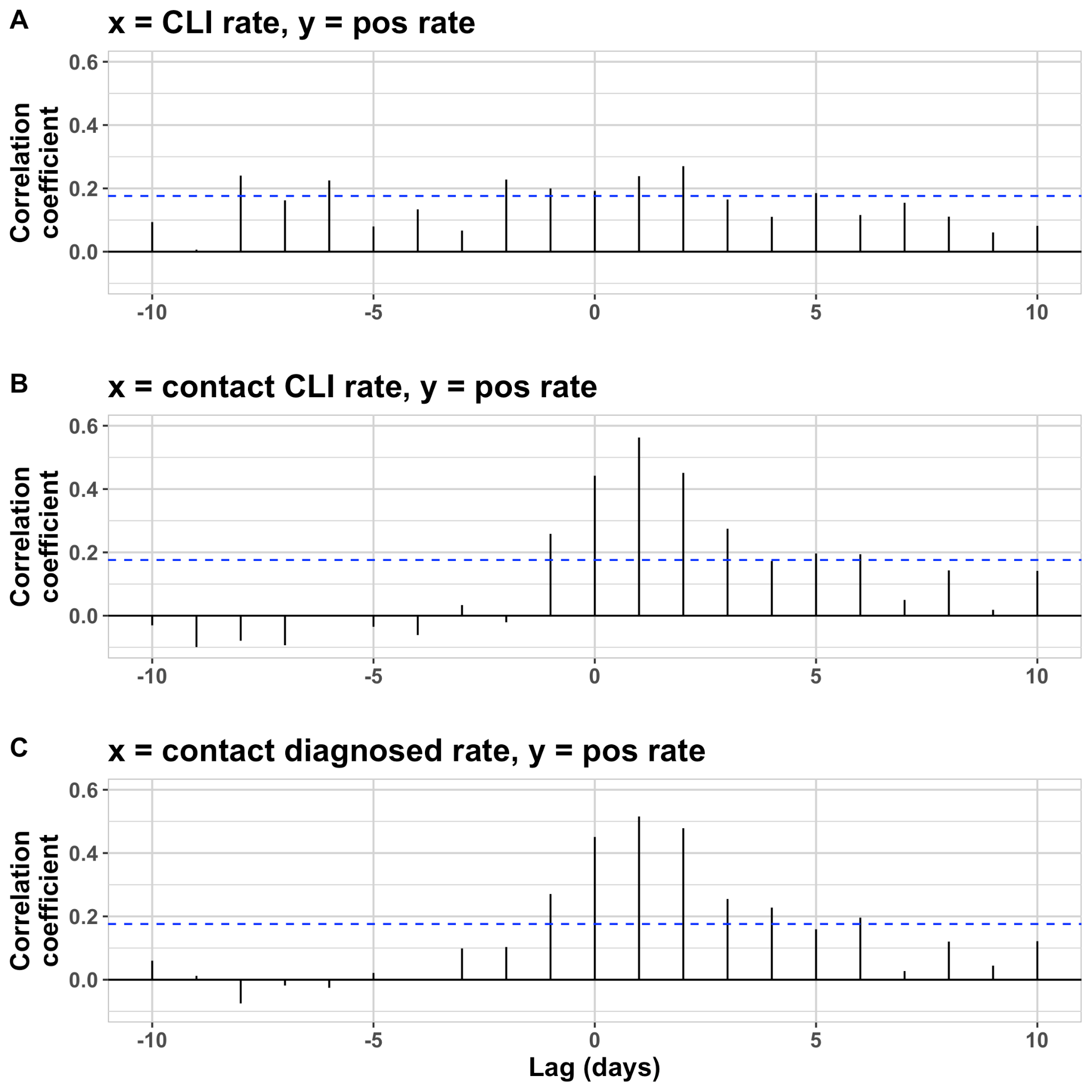


**Supplemental Figure 2.** Plot of the correlation coefficients between *x_t+h_* and *y_t_*, the rate of SARS-CoV-2-positivity, for *h* = 0, ±1 through ±10 days. When *h* < 0, the correlation coefficient quantifies the linear relationship between the *x*-variable before day *t* and the *y*-variable at *t*. If significant correlation coefficients are observed at *h* < 0, then *x* is said to lead *y*. If significant correlation coefficients are observed when *h* > 0, then *x* lags *y*. The *x*-variable is the rate of CLI (A), exposure to CLI (B), and exposure to a diagnosed case (C). Cross correlation values above the dashed blue lines indicate values are significantly different from zero.


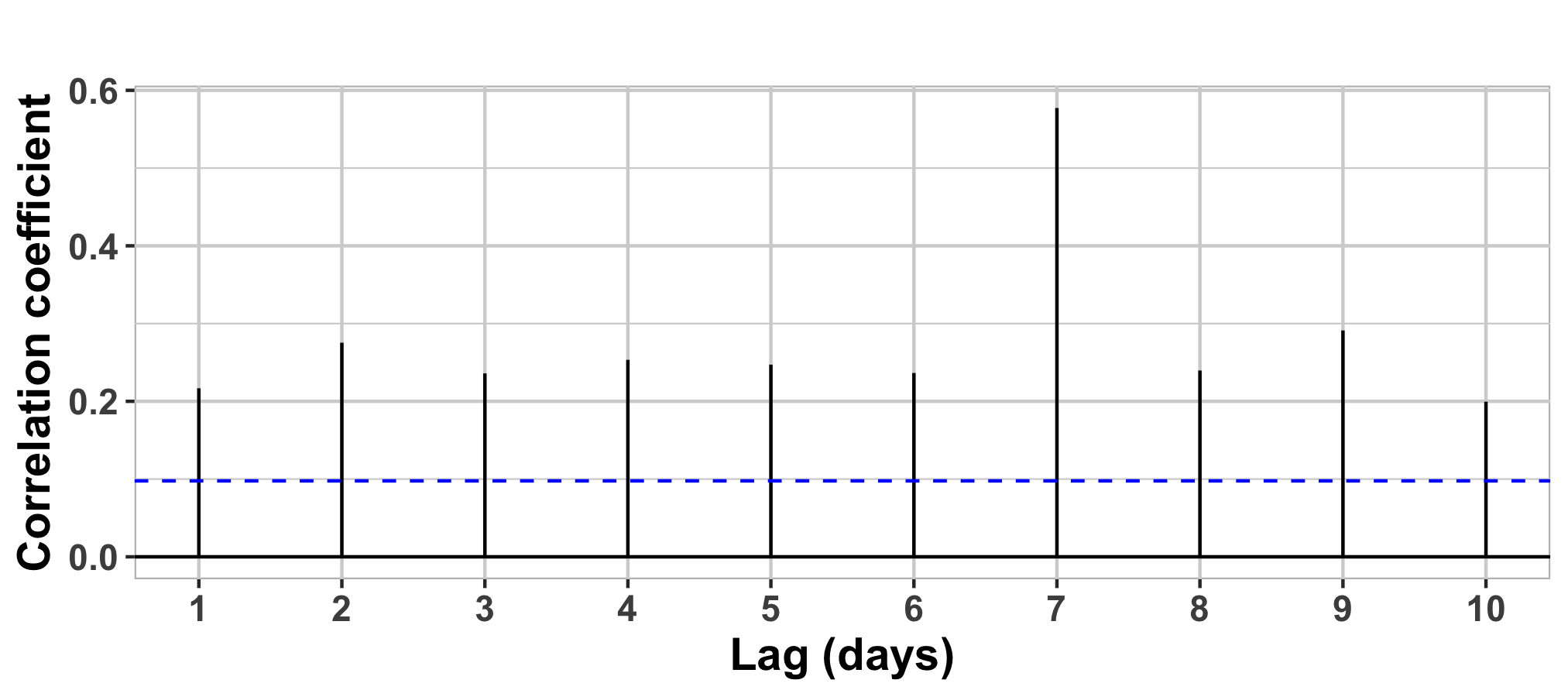


**Supplemental Figure 3.** Autocorrelation plot of correlation coefficients between SARS-CoV-2-positivity rate in Tompkins County and same variable measured days 1-10 prior. Correlation coefficients above the dashed line indicate values are significantly different from 0. All autocorrelation values are significantly different, with peaks at two and seven day lags within the first week.

# **
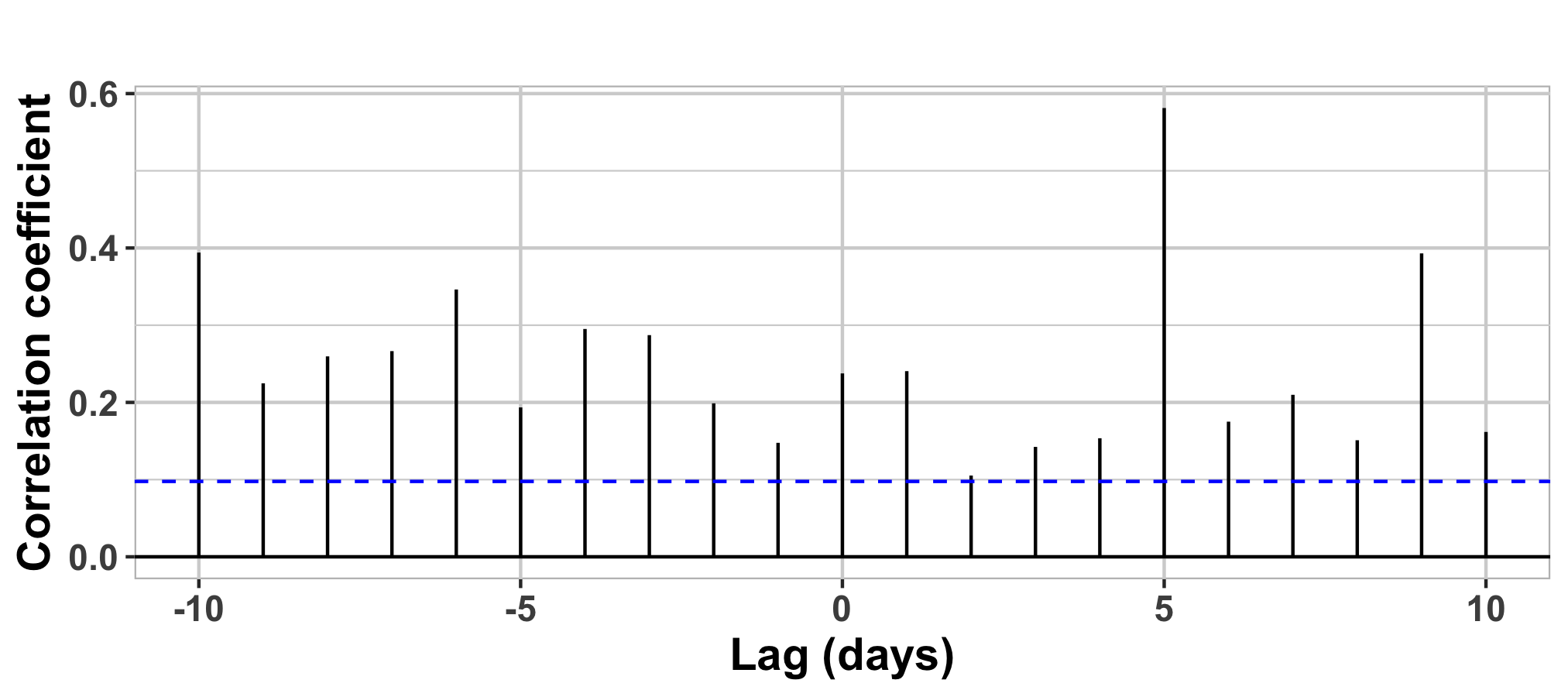
Supplemental Figure 4.** Plot of the correlation coefficients between *x_t+h_*, the CLI rate, and *y_t_*, the rate of SARS-CoV-2-positivity, for *h* = 0, ±1 through ±10 days. When *h* < 0, the correlation coefficient quantifies the linear relationship between the *x*-variable before day *t* and the *y*-variable at *t*. If significant correlation coefficients are observed at *h* < 0, then *x* is said to lead *y*. If significant correlation coefficients are observed when *h* > 0, then *x* lags *y*. Peak cross correlation coefficients occur at lag = -4, -6, and -10.

#

# **Appendix**

1. **Cornell COVID-19 Questionnaire**

Cornell’s Daily Check questions prior to February 3, 2021 included:

1. Have you ever been diagnosed/tested positive for COVID-19? (Yes/No)
   1. **If yes:** How recently were you diagnosed/tested positive for COVID-19?
2. **Before xx/xx/xx (more than 90 days ago):** student maintains “green” status and continued surveillance testing requirement
3. **Between xx/xx/xx and xx/xx/xx (less than 90 but more than 10 days ago):** student maintains “green” status and suspended surveillance testing requirement
4. **On or after xx/xx/xx (within the last 10 days):** student is given “red” status until cleared by Cornell Health to resume on campus activities
5. Have you experienced any symptoms of COVID-19 within the past 14 days? (Yes/No)
   COVID-19 symptoms include: cough, shortness of breath or difficulty breathing, fever, chills, muscle pain, sore throat, new loss of taste or smell, and (less commonly) nausea, vomiting or diarrhea. Fever is considered to be over 100°F / 38°C.
   1. **If yes:** Have you had a Cayuga Health or Cornell Health telemedicine visit for these symptoms?
      - **If no:** student is given “red” status and must meet with a Cornell Health professional
      - **If yes:** Have your symptoms worsened?
        1. **If yes:** student is given “red” status and must meet with a Cornell Health professional
        2. **If no:** Have you been cleared by Cornell Health to return to work?
           1. **If no:** student maintains “red” status
           2. **If yes:** student is given “green” status
   2. **If no:** student maintains “green” status
6. Have you knowingly been in close contact in the past 14 days with anyone who has tested positive for, or been diagnosed with, COVID-19?
   1. **If no:** student maintains “green” status
   2. **If yes:** Have you had a Cornell Health telemedicine visit for this close contact?
      - **If no:** student is given “red” status and must have a telemedicine visit before being cleared to return to campus
      - **If yes:** Have you been cleared by Cornell Health to return to work?
        1. **If no:** student maintains “red” status
        2. **If yes:** student is given “green” status
7. Have you knowingly been in close contact in the past 14 days with anyone who currently has symptoms, or had symptoms, of COVID-19?
   1. **If no:** student maintains “green” status
   2. **If yes:** Have you had a Cornell Health telemedicine visit for this close contact?
      - **If no:** student is given “red” status and must have a telemedicine visit before being cleared to return to campus
      - **If yes:** Have you been cleared by Cornell Health to return to work?
        1. **If yes:** the student is given “green” status
        2. **If no:** the student maintains “red” status

A single red status for any of the questions results in a student being placed in a “red” status overall and the student must have a telemedicine visit with Cornell Health before being cleared to return to campus.
